# Effect of topical treatment with 7.5% urea in Ichthyosis Vulgaris: A randomized, controlled, double blinded, split body study evaluating the effect of urea cream compared to the vehicle (moisturizing) cream

**DOI:** 10.1002/ski2.65

**Published:** 2021-09-14

**Authors:** I. L. H. Dorf, M. S. Lunen, U. Koppelhus

**Affiliations:** ^1^ Department of Dermatology Aarhus University Hospital Aarhus Denmark; ^2^ Department of Intern medicin Regionshospitalet Silkeborg Silkeborg Denmark; ^3^ Department of Dermatology Aarhus Universitetshospital Skejby Aarhus Denmark

## Abstract

**Background:**

Ichthyosis Vulgaris (IV) is a common genetic skin disease, characterized by dry, scaling skin and itch. Urea cream has been a central part of IV treatment for decades, but only few studies have evaluated the effect of urea compared to basic moisturizers.

**Objective:**

To evaluate the treatment effect of 7.5% urea cream compared to a basic moisturizer in patients with IV.

**Methods:**

Participants (*n* = 14) were randomized to apply the 7.5% urea cream on one body half and a basic moisturizer on the other during a study period of 4 weeks. Measuring points on participants arms and legs were evaluated at baseline and at endpoint with a patient questionnaire visual assessment scale (VAS), a clinical scoring, and electronic skin hydration analysis to assess the treatment effects.

**Results:**

On the arms, no significant differences between the two treatments were found. On the legs, however, the urea treated areas had a significantly higher decrease in SRRC score (0.7 points [95% CI: 1.1–0.3, *p* < 0.005]) and increase in hydration (32.1 μS [95% CI: 10.9‐53.2, *p* < 0.006]).

**Conclusion:**

Skin hydration improved significantly with both urea and moisturising treatment. On the legs, with most keratinization, urea was superior.

Trial registry: https://clinicaltrials.gov/ct2/show/NCT02978209?cond=Ichthyosis+Vulgaris&draw=1&rank=2.

1

**Key points**

**What is already known about this topic?**

- Ichthyosis vulgaris (IV) is the most common keratinization disorder.
- The characteristic clinical features of IV are xerosis, scaling, and itching, often accompanied by cosmetic discomfort and consequent psychosocial impairment.
- Urea containing creams are common treatments for IV.

**What does this study add?**

- A regular moisturizing regimen significantly improves hydration in the skin of IV patients.
- On the legs of IV patients, urea treatment is superior to basic moisturizer based upon a specified symptom sum score (SRRC) and hydration score.
- This study indicates that a urea containing cream may only be superior to a non‐urea‐containing moisturizer when the symptoms are severe.

**What are the clinical implications of this work?**

- The importance of a regular moisturizing regimen is confirmed in this study.
- We propose that a cream with only 7.5% instead of 10% urea minimizes the adverse effects, while maintaining a separate effect of urea. However, a separate head‐to‐head study between a 7.5% and a 10% urea cream is needed to decide if a measurable difference exist with regard to the positive outcomes of urea.

## 2 INTRODUCTION

With a prevalence between 1:250 and 1:100, ichthyosis vulgaris (IV) is the most common keratinization disorder and one of the most common genodermatoses.^1^, ^2^, ^3^ The disease is caused by loss‐of‐function variants in the *FLG* gene, which encodes profilaggrin. In normal skin the profilaggrin molecules are processed into filaggrin peptides, which bind to keratin filaments to form complexes that are essential to proper cornification of the squamous cell layer.^2^ The filaggrin is degraded into substances such as histidine, serving as natural moisturizers on the skin surface. In the skin of IV patients properly processed filaggrin is reduced (heterozygotes) or absent (homozygotes) resulting in impaired skin barrier function.^1^ IV is inherited in an autosomal semi‐dominant manner, with homozygotes being earlier and more heavily affected than heterozygotes.^3^ The penetrance of heterozygotes is 80%–95% and the phenotypic expression varies from minor symptoms to severe disease.^1^ The characteristic clinical features of IV are xerosis, scaling, and itching, often accompanied by cosmetic and sometimes ensuing psychosocial impairment. IV is associated with increased risk of atopic dermatitis and other atopic conditions.

Urea, otherwise known as carbamide, has been used to treat skin symptoms for decades and is one of the main treatments for IV. The mechanism of action of urea in skin treatment is not completely understood, but studies suggest the treatment acts both keratolytic and moisturising and may even alter gene expression of proteases in the epidermis.^4^, ^5^, ^6^ From 1968 to 2020 only five studies have evaluated the effect of (10%) urea cream by comparing with a control treatment and the treatment with urea seemed to be at least marginally better than moisturizers without urea.^5^, ^6^, ^7^, ^8^ Reported side effects include itching, erythema, pustules, and contact dermatitis.^5^, ^6^, ^7^, ^8^

The aim of the study was to investigate whether a basic moisturizing cream with 7.5% urea was more effective in treating IV than the basic cream without urea.

## 3 Participants and methods

### 3.1 Approvals

The study was approved by the Danish Scientific ethics committee.

### 3.2 Participants

The eligible participants were identified through a data extraction from the National Hospital Discharge Register of patients from Jutland and Funen with an IV diagnosis. Eligibility criteria are stated in Table 1.

**TABLE 1:** 

Eligibility criteria	
Inclusion criteria	Exclusion criteria
‐ Mild to moderate Ichthyosis vulgaris	‐ Severe ichthyosis vulgaris
‐ Age between 1 and 65 years	‐ Competing skin diseases
‐ Male or female	‐ Retinoid treatment within the last three months
	‐ Severe claustrophobia
	‐ Age below 1 year and above 65 years

All participants received both written and oral information about the study and a written consent form was signed by either participants or custodial parents.

### 3.3 Study design and intervention

The study had a randomized, controlled double‐blind, split‐body design.

DermaPharm A/S (Fårup, Denmark) manufactured two creams for the trial, which had the same base formula and only differed in urea content:

- A basic moisturizing cream.
- A basic moisturizing cream with 7.5% urea.

The tubes were randomly labelled A and B, respectively by the manufacturer blinding the trial for both participants and investigators. Ingredients shown in Table 2. Participants were randomized^9^ to apply cream ‘A’ on the right or left side of their body and cream ‘B’ on the opposite side for a four‐week study period. They were instructed to keep the creams apart and to use the creams twice daily and to abstain from other treatment during the intervention. One week before trial start participants were instructed to suspend prior moisturizing treatment to wash out. One week before baseline and endpoint examination, participants were instructed to drink a minimum of three litres of fluid each day (1–3 litres for the children depending on age) in order to be properly hydrated at the examination sessions.

**TABLE 2:** 

List of ingredients	
Cream A	Cream B
Polysorbat 80	Polysorbat 80
Cetostearylalkohol, emulgating (type B)	Cetostearylalkohol, emulgating (type B)
Paraffinoil	Paraffinoil
Glycerolmonostearat 40‐50	Glycerolmonostearat 40‐50
Methylparahydroxy‐benzoat	Methylparahydroxy‐benzoat
Glycerol 85%	Glycerol 85%
Sorbitol	Sorbitol
Water, cleaned	Water, cleaned
	Urea

## 4 OUTCOMES

### 4.1 Primary outcomes

**FIGURE 1:**
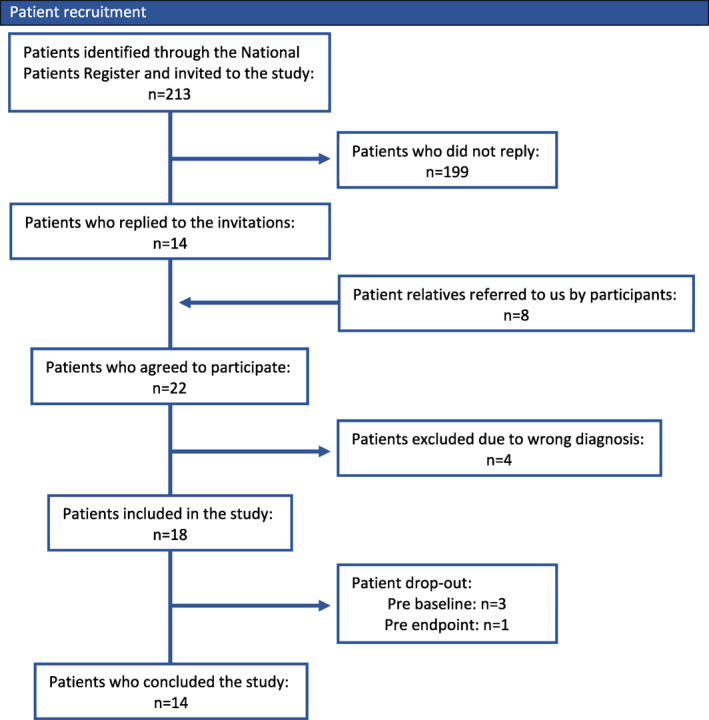
Patient recruitment

To evaluate the effect of the two treatments, the participants' skin was evaluated at baseline and at endpoint after four weeks using three primary outcomes: (1) a patient questionnaire (PQ) according to European Group on Efficacy Measurement and Evaluation of Cosmetics and other Products (EEMCO) standard,^10^ (2) a clinical scoring of skin symptoms using a specified symptom sum score (SRRC),^10^ and (3) electronic measurement consisting of trans epidermal water loss (TEWL) and hydration. The examinations were conducted at the Climate Chambers at Aarhus University,^11^ which allowed for very precise regulation of the environment. The chamber was adjusted to an ambient air humidity of 40% and a temperature of 22ºC, providing optimal conditions for electronic analysis of TEWL and hydration.^12^ At baseline and endpoint, six measuring points were defined by drawing circles of ∅ 2–3 cm with numbers referring to specific areas on the participants' arms and legs (see Table 3 and Figure 1). All markings for each participant were photographed at baseline to ensure that exactly the same areas of the participant's body could be precisely re‐established and re‐measured throughout the study. Participants were dressed in shorts and t‐shirts to prevent sweating and occlusion of the skin, and both the electric instruments for skin evaluation and participants were acclimatized for minimum one hour prior to measurements.

**TABLE 3:** 

Measuring areas		
	Right side, numbering	Left side, numbering
Arm, distal	1	3
Arm, proximal	2	4
Leg	5	6

#### 4.1.1 Patient questionnaire

The PQ was developed according to guidelines for the assessment of ichthyosis made by the EEMCO,^10^ On a visual analogue scale (VAS) with the endpoints marked with ‘no xxx’ and ‘worst xxx ever’, participants evaluated five parameters for each of the measuring areas. The parameters were dryness, scaling, itching, stinging, and pain. The markings on the VAS were converted to a score from 0 to 10 for each parameter. Based on the individual scores, a combined score was calculated for each measuring point.

#### 4.1.2 SRRC score

The SRRC score is another assessment tool from the EEMCO group,^10^ in which an expert evaluates the participants' skin. All measuring points were scored on a scale from 0 to 4 (0 = absent, 1 = slight, 2 = moderate, 3 = severe, and 4 = extreme) on the four symptoms scaling, roughness, redness, and cracks/fissures, and the sum (SRRC score) was calculated for each measuring point.

#### 4.1.3 Electronic skin analysis

The electronic skin analysis was performed using the DermaLab^®^ system (Cortex Technology) with the TEWL and hydration probes. TEWL is widely recognized as a valid non‐invasive in vivo measure for skin barrier function, while hydration measured as conductance has been shown to be a relevant measure for especially superficial stratum corneum water content.^13^, ^14^, ^15^ TEWL is measured as g/m^2^/h and hydration is measured in microSiemens (μS). At each measuring point TEWL was measured five times and hydration eight times and, based on the individual measurements, mean TEWL, and hydration values were calculated for each measuring point. Both methods are reviewed in a separate method study (manuscript in preparation).

### 4.2 Secondary outcomes

At the endpoint examination, the participants answered a questionnaire about their experience with the creams. On a VAS, participants answered questions about the smell of the cream, how easy the cream was to apply, and how the skin felt five minutes after application. The VAS indications were converted to scores from 0 to 5. The participants were asked to describe the effect after one month and any side effects they experienced. Finally, the participants were asked to mark their favourite cream or to mark both if they felt equally good or bad.

#### 4.2.1 Statistical analysis

All data was collected and managed using REDcap, hosted at Aarhus University.^16^

Sample size calculations were performed with G*power based on assumptions about the difference in SRRC score from baseline to endpoint between the two treatment groups. The following assumptions were made on the basic of results from a study by Tadini et al.^8^: difference in mean difference between baseline and endpoint of at least two points, Standard deviation (SD) (urea): 2, SD (control): 2.5 and correlation: 0.5. At a power of 90% and an α‐level of 0.05, a sample size of 17 participants in each treatment group was estimated to be necessary. Because of the split body design, each participant belongs to both treatment groups, and thus 17 participants in total were needed. To take possible drop out into account, the intention was to include 20 participants.

Statistical analyses were performed with Stata (version 15.1, StataCorp, College Station). QQ‐plots were used to evaluate normal distribution of continuous variables. A *t*‐test and the Wilcoxon signed‐rank test were used to compare baseline values of the primary outcomes between the two treatment groups. A paired *t*‐test and the Wilcoxon signed‐rank test were applied to compare the difference in mean difference between endpoint and baseline values for the three primary outcomes between the two treatment groups for both the entire body halves and the individual measuring points. The secondary outcomes were evaluated using Wilcoxon signed‐rank test. Throughout the analysis, a *p*‐value of <0.05 was considered significant.

## 5 RESULTS AND ANALYSIS

### 5.1 Participants and baseline characteristics

Out of 213 eligible participants, 14 subjects responded to the invitation and an additional eight were referred to the investigators by participants. The 22 subjects were examined and four were excluded due to wrong diagnosis, which in all four cases were X‐linked ichthyosis. Eighteen subjects ended up being included, but three failed to appear at the baseline examination for reasons unknown and one participant could not attend endpoint examination. Thus, 14 IV‐patients ended up completing the study. A flowchart of participant inclusion is shown in Figure 1. Participant characteristics at baseline are shown in Table 4.

**TABLE 4:** 

Participant characteristics at baseline	
Number of participants, *N*	14
Age (years), Mean (SD)	26 (20)
Age (years), range	4‐60
Sex (male/female)	6/8

### 5.2 Primary outcomes

#### 5.2.1 Exclusion of TEWL as an outcome measure

During the study, we noticed significant inconsistencies in the TEWL measurements. These inconsistencies need further analysis and interpretation and possibly further experiments in order to be explained. Therefore, the TEWL results will not be included in this report. The problems with TEWL are addressed in a separate method study (manuscript in preparation).

#### 5.2.2 Baseline values

At baseline, no significant difference was found between sides later treated with either cream A or cream B in any of the primary outcomes (Table 5). Each measuring point was analysed individually as well, but no difference between sides was found for either of the points.

**TABLE 5:** 

Baseline values of primary outcomes^a^					
	Cream A, mean (95% CI) [SD]	Cream B, mean (95% CI) [SD]	Difference, mean (95% CI) [SD]	*T*‐test, *P*‐value	Wilcoxon signed‐rank test, *p*‐value
Number of participants, n	14	14	0	‐	‐
Patient questionnaire score^b^	10.5 (8.2–12.7) [3.9]	11.2 (8.6–13.8) [4.5]	−0.7 (−1.8–0.3) [1.9]	0.17	0.06
SSRC‐score	2.7 (1.8–3.6) [1.5]	2.7 (1.8–3.6) [1.6]	−0.05 (−0.2–0.1) [0.2]	0.34	0.34
Hydration	106.0 (90.3–121.7) [27.2]	105.5 (91.4–119.5) [24.4]	0.5 (−8.0–9.1) [14.8]	0.89	0.93

^a^ The participant who dropped out after baseline was excluded from this analysis. The same analysis, including the participant, led to the same overall conclusions.

^b^ The Wilcoxon signed‐rank test for the proximal arm yielded a *p*‐value of 0,03. *T*‐test for this point had a *p*‐value of 0,21.

Baseline values for the arms and legs were compared and for all parameters the legs were significantly more severely affected than the arms, in regard to having higher patient questionnaires‐scores, higher SRRC scores and lower hydration. The individual measuring points on the arms only differed significantly from each other in hydration (results shown in Tables 6 and 7).

**TABLE 6:** 

Baseline values of primary outcomes^a^					
	Cream A, mean (95% CI) [SD]	Cream B, mean (95% CI) [SD]	Difference, mean (95% CI) [SD]	*T*‐test, *P*‐value	Wilcoxon signed‐rank test, *p*‐value
Number of participants, *n*	14	14	0	‐	‐

Baseline values for entire body halves					
Patient questionnaire score	10.5 (8.2–12.7) [3.9]	11.2 (8.6–13.8) [4.5]	−0.7 (−1.8–0.3) [1.9]	0.17	0.06
SSRC‐score	2.7 (1.8–3.6) [1.5]	2.7 (1.8–3.6) [1.6]	−0.05 (−0.2–0.1) [0.2]	0.34	0.34
Hydration	106.0 (90.3–121.7) [27.2]	105.5 (91.4–119.5) [24.4]	0.5 (−8.0–9.1) [14.8]	0.89	0.93

Baseline values for distal arms					
Patient questionnaire score	6.0 (4.2–7.8) [3.1]	6.5 (4.1–8.9) [4.1]	−0.5 (−1.7–0.8) [2.2]	0.44	0.75
SSRC‐score	1.7 (0.6–2.8) [1.9]	1.7 (0.6–2.8) [1.9]	0 (0‐0) [0]	1.0	1.0
Hydration	113.2 (93.9–132.5) [33.4]	115.5 (98.1–132.9) [30.1]	−2.3 (−13.9–9.3) [20.1]	0.67	0.78

Baseline values for proximal arms					
Patient questionnaire score	7.1 (4.6–9.5) [4.3]	8.4 (4.8–12.1) [6.3]	−1.4 (−3.6–0.9) [3.9]	0.21	0.03
SSRC‐score	1.5 (0.6–2.4) [1.5]	1.5 (0.7–2.4) [1.5]	−0.07 (−0.2–0.1) [0.3]	0.34	0.34
Hydration	147.3 (124.9–169.8) [38.8]	149.0 (123.5–174.5) [44.1]	−1.7 (−15.8–12.5) [24.5]	0.80	0.88

Baseline values for legs					
Patient questionnaire score	18.3 (13.8–22.9) [7.9]	18.7 (14.0–23.4) [8.1]	−0.4 (−1.2–0.4) [1.4]	0.31	0.40
SSRC‐score	4.8 (3.7–5.9) [1.8]	4.9 (3.8–6.0) [1.9]	−0.07 (−0.3–0.2) [0.5]	0.58	0.56
Hydration	57.5 (44.0–71.0) [23.4]	51.9 (43.6–60.2) [14.5]	5.6 (−2.6–13.9) [14.3]	0.17	0.27

^a^ The participant who dropped out after baseline was excluded from this analysis. The same analysis, including the participant, led to the same overall conclusions.

**TABLE 7:** 

Baseline values for distal arms compared to baseline values of proximal arms					
	Baseline values distal arms, mean (95% CI) [SD]	Baseline values proximal arms, mean (95% CI) [SD]	Difference, mean (95% CI) [SD]	*T*‐test, *P*‐value	Wilcoxon signed‐rank test, *p*‐value
Patient questionnaire score	6.2 (4.2–8.3) [3.5]	7.7 (4.8–10.6) [5.0]	−1.5 (−3.4–0.3) [3.2]	0.10	0.40
SSRC‐score	1.7 (0.6–2.8) [1.9]	1.5 (0.7–2.4) [1.5]	0.2 (−0.4–0.8) [1.1]	0.54	0.83
Hydration	114.3 (96.9–131.8) [30.2]	148.2 (125.2–171.1) [39.7]	−33.8 (−50.5–−17.2) [28.8]	0.0007	0.004

Baseline values for distal arms compared to baseline values of legs					
	Baseline values distal arms, mean (95% CI) [SD]	Baseline values proximal arms, mean (95% CI) [SD]	Difference, mean (95% CI) [SD]	*T*‐test, *P*‐value	Wilcoxon signed‐rank test, *p*‐value
Patient questionnaire score	6.2 (4.2–8.3) [3.5]	18.5 (13.9–23.1) [8.0]	−12.3 (−16.8–−7.8) [7.8]	0.0001	0.001
SRRC score	1.7 (0.6–2.8) [1.9]	4.8 (3.7–5.9) [1.9]	−3.1 (−4.1–−2.1) [1.7]	0.0000	0.001
Hydration	114.3 (96.9–131.8) [30.2]	54.7 (44.3–65.1) [18.1]	59.6 (43.7–75.6) [27.6]	0.0000	0.001

Baseline values for proximal arms compared to baseline values of legs					
	Baseline values distal arms, mean (95% CI) [SD]	Baseline values proximal arms, mean (95% CI) [SD]	Difference, mean (95% CI) [SD]	*T*‐test, *P*‐value	Wilcoxon signed‐rank test, *p*‐value
Patient questionnaire score	7.7 (4.8–10.6) [5.0]	18.5 (13.9–23.1) [8.0]	−10.8 (−16.0–−5.6) [9.0]	0.0006	0.001
SRRC score	1.5 (0.7–2.4) [1.5]	4.8 (3.7–5.9) [1.9]	−3.3 (−4.2–−2.3) [1.7]	0.0000	0.0009
Hydration	148.2 (125.2–171.1) [39.7]	54.7 (44.3–65.1) [18.1]	93.5 (72.3–114.7) [36.7]	0.0000	0.001

Abbreviation: SRRC, specified symptom sum score.

#### 5.2.3 Effect after four weeks of treatment

Considering the patient questionnaire (PQ), we found no statistically significant difference in effect of the two treatments. Only the basic cream showed a statistically significant effect from baseline to endpoint (−3.4 points (95% CI: −6.3–−0.4)). In both treatment groups the SRRC score decreased significantly from baseline to endpoint with the mean difference being −1.3 points (95% CI: −1,9–−0.6) for the urea treatment and −1 point (95% CI: −1.7–−0.3) for the basic treatment. The difference in effect between the two treatments was statistically significant with −0.3 points (95% CI: −0.5–−0.06, *p* < 0.03). The total hydration values increased significantly from baseline to endpoint in both groups with a mean difference in hydration values of 83.3 μS (95% CI: 55.1–111.5) for the urea treatment and 63.7 μS (95% CI: 36.9–90.5) for the basic (vehicle) cream. The hydration increase with the urea treatment was significantly larger than for the basic cream (19.6 μS [95% CI: 8.6–30.5, *p* < 0.006]).

When analysing the measuring points individually, results that were more ambiguous were obtained.

For both measuring points on the arms, no significant difference between the effects of the two treatments for either of the outcome measures was found. Hydration score was significantly increased for both urea treatment and basic treatment for the distal arm (83.3 μS (95% CI: 55.1–111.5) and 63.7 μS (95% CI: 36.9–90.5), respectively) as well as the proximal arm (94.4 μS (95% CI: 62.7–126.0) and 83.0 μS (95% CI: 37.7–128.3), respectively). The SRRC score for the urea treated proximal arm significantly decreased with 0.9 points (95% CI: 1.7–0.05) from baseline to endpoint. Apart from this, no significant effects were seen for either treatment on the arm measuring points.

For the legs, statistically significant differences from baseline to endpoint were seen with both treatments for all three outcome measures. PQ scores decreased by 8.2 points (95% CI: 13.4–3.1) with urea‐treatment and by 7.8 points (95% CI: 12.8–2.8) when treated with basic cream, while the SRRC scores decreased by 2.2 points (95% CI: 3.0–2.4) and 1.5 points (95% CI: 2.3–0.7) for urea treatment and basic cream treatment, respectively. Finally, the hydration scores increased by 76.6 μS (95% CI: 44.3–108.8) with urea treatment and by 44.5 μS (95% CI: 27.5–61.5) with basic cream treatment. Statistically significant difference between the effect of the two treatments were found for both SRRC score and hydration score. Areas treated with urea had 0.7 points (95% CI: 1.1–0.3, *p* < 0.005) larger decrease in SRRC score than seen in areas treated with basic cream, while a 32.1 μS (95% CI: 10.9–53.2, *p* < 0.005) larger increase in hydration was observed in urea‐treated areas compared to areas treated with basic cream. There was no significant difference between the two treatments with respect to the PQ. All results are shown in Table 8.

**TABLE 8:** 

Effect of treatments after 4 weeks of treatment					
	Urea cream, mean difference (endpoint−baseline) (95% CI) [SD]	Basic cream, mean difference (endpoint−baseline) (95% CI) [SD]	Difference, mean difference (Urea‐Basic) (95% CI) [SD]	Paired *T*‐test, *p*‐value	Wilcoxon signed‐rank test, *p*‐value
Number of participants, *n*	14	14	0	‐	‐

Effect on entire body halves					
Patient questionnaire score	−3.1 (−6.3–0.1) [5.5]	−3.4 (−6.3–−0,4) [5.1]	0.3 (−1.2–1.8) [2.6]	0.71	0.47
SRRC score	−1.3 (−1.9–−0.6) [1.07]	−1 (−1.7–−0.3) [1.3]	−0.3 (−0.5–−0.06) [0.4]	0.02	0.03
Hydration score	83.3 (55.1–111.5) [48.8]	63.7 (36.9–90.5) [46.5]	1.6 (8.6–30.5) [18.9]	0.002	0.006

Effect on distal arms					
Patient questionnaire score	0.03 (−2.6–2.6) [2.5]	−0.2 (−2.7–2.3) [4.3]	0.3 (−1.1–1.6) [2.3]	0.68	0.90
SRRC score	−0.9 (−1.7–−0.05) [1.4]	−0.8 (−1.7–0.1) [1.5]	−0.1 (−0.2–0.1) [0.3]	0.33	0.31
Hydration score	78.9 (41.2–116.5) [65.2]	63.6 (29.5–97.8) [59.2]	15.3 (−9.0–39.5) [41.9]	0.20	0.18

Effect on proximal arms					
Patient questionnaire score	−1.1 (−4.3–2.2) [5.6]	−2.0 (−5.4–1.4) [5.9]	0.9 (−1.4–3.3) [4.1]	0.40	0.63
SRRC score	−0.7 (−1.5–0.1) [1.3]	−0.7 (−1.5–0.1) [1.4]	0 (−0.3–0.1) [0.6]	1.0	1.0
Hydration score	94.4 (62.7–126.0) [54.9]	83,0 (37,7; 128,3) [78,4]	11.4 (−16.4–39.1) [48.1]	0.39	0.22

Effect on legs					
Patient questionnaire score	−8.2 (−13.4– −3.1) [8.9]	−7.8 (−12.8–−2.8) [8.7]	−0.4 (−2.6–1.8) [3.8]	0.68	0.98
SRRC score	−2.2 (−3.0–−2.4) [1.4]	−1.5 (−2.3–−0.7) [1.4]	−0.7 (−1.1–−0.3) [0.7]	0.003	0.005
Hydration score	76.6 (44.3–108.8) [55.83]	44.5 (27.5–61.5) [29.4]	32.1 (10.9–53.2) [36.7]	0.006	0.0052

Abbreviation: SRRC, specified symptom sum score.

### 5.3 Secondary outcomes

On the four parameters in the VAS questionnaire on the participants' experience with the creams, participants generally scored both of the creams as medium to good. The two creams differed only in how easy they were to apply, with the basic cream being perceived as easier to apply to the skin (*p* = 0.01). Results are shown in Table 9. No significant difference in weight after the four weeks were measured between cream A and B.

**TABLE 9:** 

Results for questionnaire about participants' experience with the creams			
	Urea cream, median (range)	Basic cream, median (range)	Wilcoxon signed‐rank test, *p*‐value
Number of participants, *n*	14	14	‐
Smell in container (0 = good, 2.5 = neutral, 5 = bad)	2.6 (0.5–3)	2.5 (0.4–3.5)	0.67
Smell on skin (0 = good, 2.5 = neutral, 5 = bad)	2.5 (2.4–3.8)	2.5 (0.8–3.6)	0.87
Application on skin (0 = very easy, 2.5 = neutral, 5 = very bad)	2.2 (0.2–3.1)	0.7 (0.3–2.4)	0.01
Five min. skin feel (0 = cream quickly absorbed, 5 = sticky)	1.15 (0.3–4.5)	1.5 (0.3–4.5)	0.77

Most participants described the results of both treatments to be a reduction of dryness and softer skin. Two participants reported a reduction of scaling on both sides treated.

Four participants reported unwanted effects during the study. At the start of the treatment, one participant experienced scaling of the fingertips on both sides treated, but this quickly disappeared. One participant developed red spots under her breasts on both sides. She stopped applying the creams on that location and the rash disappeared. Two participants with concomitant atopic dermatitis experienced slight irritation and itch in eczematous regions (no side difference). Thus, no unwanted effects were reported specifically for one of the treatments.

Five participants preferred cream ‘A’ (the urea cream), six participants preferred ‘B’ (i.e., the basic cream) and three participants preferred ‘A and B’.

## 6 DISCUSSION

Treatment for IV is only symptomatic and aims at increasing the water content in the skin and reduce hyperkeratosis.^17^ In the present study, we evaluated whether a moisturizer containing 7.5% urea was superior in treating IV symptoms compared to the basic vehicle moisturizer without urea.

At baseline, the legs were significantly more affected than the arms on all parameters measured. Analysis of entire body halves resulted in statistically significant differences between treatment effects on both SRRC score and hydration score. When analysing the different measuring points individually, this significant difference turned out only to be the result of highly significant differences found on the legs. No difference between the treatments were seen on the arms. This indicates that urea may only be superior to moisturizers when the symptoms are more severe.

With regards to the hydration score, the magnitude of the difference in increase (32.1 μS [95% CI: 10.9; 53.2]) between the two treatments was unexpected. At baseline, mean hydration score on the legs was 54.7 μS (95% CI: 44.3; 65.1), and in this context a difference of 32.1 μS should be considered relevant. Significant increases (ranging from 45 to 94 μS) were seen for all measuring points with both treatments. This was the only parameter significantly affected by treatments on both arms and legs, which may suggest that electronic measurement of hydration is more sensitive to changes in the skin than clinical assessment and self‐evaluation.

We found that both the urea and the moisturizing creams were well tolerated. Many of the reported adverse effects of urea containing moisturizers are seen in patients treated with 10% urea cream,^8^ and we propose that a cream with 7.5% urea minimizes the adverse effects, while maintaining a separate effect of urea. However, a separate head‐to‐head study between a 7.5% and a 10% urea cream is necessary to prove this hypothesis and to decide if a measurable difference exist with regard to the positive outcomes of urea.

Based on our results, we cannot completely decide whether urea treatment is generally better than a basic moisturizer. However, we only included patients with mild to moderate disease, and it is possible that the isolated effect of urea would be more pronounced treating IV patients with more severe disease as indicated by the effect on the legs.

### 6.1 Strengths

The split‐body design is favoured by the participants serving as their own controls and thereby avoiding any kind of bias to the two treatment “groups”. Blinding was maintained throughout the study for both participants and investigators. The climate chambers provided a highly controlled environment for the electronic measurements.

### 6.2 Limitations

We failed to include the appropriate number of patients according to our sample size calculations, which may entail that our power has become insufficient, and thus, our risk of making a type II error has increased. However, in our initial sample size calculation we made the same assumptions with a power of 80%, and this resulted in an estimated sample size of 13 participants. 80% is still a fair power level and we consider the risk of type II errors in our study to be minimal. An additional problem with the sample size is that it was calculated on the basis of assumptions about only one of the outcome measures, the SRRC score. Therefore, reservations should be made about concluding statements about the other outcome measures.

## 7 CONCLUSION

Skin hydration improves significantly in IV patient with both urea treatment and basic moisturizing treatment, indicating that a regular moisturizing regime in itself is a favourable treatment for the symptoms of IV. On the legs, which were significantly more affected than the arms, SRRC scores and hydration scores were significantly more improved on the urea‐treated side compared to the side only treated with basic moisturizer. Both treatments were equally well tolerated by the participants.

## CONFLICTS OF INTEREST

Uffe Koppelhus and DermaPharm A/S are collaborating on the cosmetics brand MDerma.

## AUTHOR CONTRIBUTIONS

**I. L. H. Dorf:** Conceptualization; Data curation; Formal analysis; Funding acquisition; Investigation; Methodology; Project administration; Resources; Software; Supervision; Validation; Visualization; Writing – original draft; Writing – review & editing. **M. S. Lunen:** Conceptualization; Data curation; Formal analysis; Funding acquisition; Investigation; Methodology; Project administration; Resources; Software; Supervision; Validation; Visualization; Writing – original draft; Writing – review & editing. **U. Koppelhus:** Conceptualization; Data curation; Formal analysis; Funding acquisition; Investigation; Methodology; Project administration; Resources; Software; Supervision; Validation; Visualization; Writing – original draft; Writing – review & editing.

## Data Availability

Data are available by contacting the corresponding author inger.dorf@live.com.
